# Unravelling cyberbullying among Egyptian adolescents: the protective influence of self-control and moral identity with gender and socioeconomic dynamics

**DOI:** 10.1186/s40359-025-02630-2

**Published:** 2025-04-16

**Authors:** Basma Elsayed Mohamed Othman, Shymaa Mahmoud Zedan Abdelhai, Mohammed Ibrahim Touni Ibrahim, Alaa Eldin Moustafa Hamed, Abeer Moustafa Barakat, Essam Eltantawy Elsayed

**Affiliations:** 1 Psychiatric Mental Health Nursing Faculty of Nursing, Cairo University, Cairo, Egypt; 2 Psychology Faculty of Humanities Studies, Al Azhar University, Cairo, Egypt; 3 Faculty of Nursing, Jerash University, Jerash, Jordan; 4 Community Health Nursing at Minia University Hospitals, Minia University, Minia, Egypt; 5 Psychiatric and Mental Health Nursing, Faculty of Nursing, Helwan University, Cairo, Egypt; 6 Maternal and Newborn Health Nursing, Faculty of Nursing, Helwan University, Cairo, Egypt; 7 Department of Nursing, College of Applied Medical Sciences, Hafr Albatin University, Hafr Albatin, Saudi Arabia

**Keywords:** Cyberbullying, Self-control, Moral identity, Adolescents, Psychological well-being, Nursing interventions

## Abstract

**Background:**

Cyberbullying presents a significant challenge for adolescents, leading to severe psychological and social consequences. This study explores the relationships between cyberbullying, self-control, and moral identity among Egyptian school students, considering gender and socioeconomic factors from a nursing perspective.

**Methods:**

A cross-sectional, descriptive correlational study was conducted among 304 preparatory and secondary school students aged 12–20 years in Egypt. Data were collected through a validated online questionnaire incorporating standardized tools to assess cyberbullying involvement, self-control, and moral identity. Statistical analyses included correlation and regression to examine the relationships between study variables. Ethical approval was secured, and informed consent was obtained from participants and legal guardians.

**Results:**

Findings revealed that 78.6% of students had low cyberbullying involvement, 58.6% exhibited moderate self-control, and 78.6% demonstrated high moral identity. Self-control showed a significant negative correlation with cyberbullying (*r* = -0.32, *p* < 0.001) and emerged as a strong predictor of lower cyberbullying engagement (β = -0.287, *p* < 0.001). Although moral identity did not directly predict cyberbullying, gender moderated its influence (β = -0.221, *p* = 0.006). Socioeconomic status did not significantly mediate the relationships among self-control, moral identity, and cyberbullying.

**Conclusion:**

Self-control serves as a critical protective factor against cyberbullying among adolescents, while moral identity’s role appears to be gender dependent. These findings emphasize the necessity of targeted interventions in school health programs to enhance self-regulation and ethical awareness among students. Psychiatric and school nurses should integrate cyberbullying prevention strategies into educational initiatives, reinforcing self-control development and ethical reasoning. Future research should further explore psychological and social determinants of cyberbullying and evaluate the effectiveness of nursing-led interventions in adolescent populations.

**Clinical trial number:**

Not applicable.

## Introduction

Cyberbullying is a form of bullying that occurs through electronic devices, affecting individuals or groups. It is characterized by repetitive behavior and intentional aggression aimed at harming the victim. There are three main roles in cyberbullying: victims, perpetrators (bullies), and individuals who experience both victimization and perpetration. Research suggests that many victims eventually become bullies themselves [1]. Previous studies indicate that more than 75% of children and adolescents have encountered cyberbullying [2].

Cyberbullying among adolescents and young adults is a significant public health concern, closely linked to adolescent behavior, mental health, and development [3]. The increasing global adoption of the Internet and the widespread use of social media have exacerbated this issue, exposing many children and adolescents to cyberbullying and online victimization. Unlike traditional bullying, cyberbullying transcends physical boundaries, as virtual environments eliminate spatial and temporal constraints, allowing perpetrators to target victims regardless of geographical limitations [4].

The psychological impact of cyberbullying is particularly concerning, as it compromises personal privacy and induces distress. Its effects may be even more severe than traditional bullying, given that perpetrators can act anonymously and target individuals at any time and in any place [5]. To understand the mechanisms driving cyberbullying behavior, the Ecological Systems Theory provides a comprehensive framework. This theory posits that multiple risk factors across different ecological levels self, family, school, peers, and employment shape adolescent behavior. These domains interact dynamically, influencing the likelihood of delinquent actions by shaping underlying motivations and behavioral patterns [6].

Among these risk factors, individual self-regulation mechanisms, particularly self-control, play a pivotal role in mitigating cyberbullying. As adolescents navigate social and environmental influences, their ability to regulate impulses and align their behavior with moral and societal expectations becomes crucial [7]. Baumeister et al., (2007) define self-control as the ability to regulate one’s responses in accordance with moral principles, societal expectations, and long-term goals [8]. Similarly, Denson et al., (2011) highlights its role in resisting violent impulses [9]. Research further suggests that provocation followed by rumination weakens self-control and heightens aggression, whereas strong self-control reduces aggressive behavior. These effects are regulated by prefrontal cortical mechanisms, which suppress aggressive tendencies and promote self-regulation [10].

Given the significance of self-control in shaping behavior, it is essential to understand the key factors that contribute to its development. Three fundamental components influence an individual’s ability to exercise self-control: self-discipline, habit-breaking, and temptation resistance. Habit-breaking refers to the ability to regulate thoughts, emotions, and behaviors in accordance with societal norms. Self-discipline involves the capacity to act against habitual tendencies when necessary. Lastly, temptation resistance is the ability to recognize and dismiss temptations, preventing impulsive actions [11].

While self-control is a key determinant of cyberbullying behavior, moral identity also plays a crucial role. Cyberbullying differs from traditional bullying in that it offers greater anonymity, invisibility of victims, and delayed gratification, which may influence how moral disengagement mechanisms operate [12]. However, moral identity is defined as the extent to which morality is central to one’s self-concept acts as a protective factor. A strong moral identity fosters prosocial actions while deterring unethical behaviors, such as academic dishonesty and violent conduct [13]. Additionally, various individual and environmental factors parenting styles, school environment, peer associations, moral capacity, self-control, social learning, psychological strain, and perceived deterrents—contribute to cyberbullying perpetration [14, 15].

The relationship between moral identity, self-control, and cyberbullying can be explained through cognitive and social cognitive theories, which emphasize the role of individual perceptions and beliefs in shaping moral behavior. Piaget’s theory of moral reasoning suggests that ethical behavior is predicted by moral judgment, while Kohlberg’s stages of moral development highlight the influence of moral reasoning on decision-making and fairness. Social cognitive theory further explains how self-regulation mechanisms are shaped by an individual’s cognitive goals and acquired knowledge, which in turn influence behavior across various situations [16, 17].

Moral identity develops through an ongoing evolutionary process that begins in childhood and continues into adulthood. Throughout this process, an individual’s moral values and beliefs shape their behavior across different contexts [18, 19]. Studies indicate that attitude is a key predictor of cyberbullying activity, linking students’ violent intentions, behavioral control, subconscious norms, and attitudes toward school violence [20, 21]. Some scholars advocate for enhancing students’ moral consciousness, critical thinking, and resilience to strengthen their intent for moral action and reduce the likelihood of cyberbullying [22].

Building on these findings, it is essential to explore the roles of moral identity and self-control in mediating cyberbullying among school students. Although moral identity has been linked to traditional bullying, its role in cyberbullying remains underexplored. Given the aggressive nature of cyberbullying, it is reasonable to hypothesize that adolescents with stronger moral identities may exhibit lower rates of cyberbullying perpetration [23, 24].

Cyberbullying is a serious public health concern among adolescents, leading to severe psychological, emotional, and social consequences. While external factors like peer influence and parental involvement have been extensively studied, the internal psychological mechanisms shaping adolescents’ online moral decision-making remain underexplored. Moral identity, though linked to ethical behavior and traditional bullying, has an unclear direct influence on cyberbullying. Similarly, while self-control helps regulate aggression, its interaction with moral identity in preventing cyberbullying is insufficiently studied. Despite cyberbullying rates ranging from 20 to 40% globally, with similar trends in the Middle East, research in Egypt remains limited.

To address these gaps, this cross-sectional, descriptive correlational study systematically examines the relationship between cyberbullying, self-control, and moral identity among Egyptian school students. Grounded in self-regulation and social cognitive theories, it investigates how self-control and moral identity predict cyberbullying behavior, hypothesizing that lower self-control and weaker moral identity are associated with higher cyberbullying tendencies. By considering gender and socioeconomic factors, this study aims to provide insights that will inform school health programs, mental health policies, and educational interventions to enhance adolescents’ self-regulation and moral reasoning, ultimately reducing cyberbullying.

## Methods

### Aim of the study

This study aims to examine the relationship between cyberbullying, self-control, and moral identity among Egyptian school students, considering the moderating role of gender and the mediating influence of socioeconomic status.

### Setting and study design

This study was conducted in preparatory and secondary schools in Dakahlia Governorate, Egypt. A descriptive correlational design was adopted to investigate the relationship between cyberbullying, self-control, and moral identity among school students. A cross-sectional correlational design was employed, adhering to the principles outlined in the Strengthening the Reporting of Observational Studies in Epidemiology (STROBE) guidelines [25].

### Sample size and study sampling

The study employed a purposive sampling technique to recruit 304 preparatory and secondary school students aged 12–20 years from governmental schools in Dakahlia Governorate, Egypt, ensuring the inclusion of students actively engaged in digital communication, a key factor in studying cyberbullying behaviors. Participants were selected based on specific inclusion criteria: being within the 12–20 age range, enrolled in preparatory or secondary school, active internet and social media users, and willing to participate, with informed consent obtained from students and legal guardians (for minors). The sample size was determined using a statistical equation, considering a 5% level of significance and an 80% study power, based on data from existing literature.

The sample size was calculated using the following formula:

$$\mathrm{n}=\frac{\left(\mathrm{z} 1-\frac{\alpha}{2}\right)^2 \cdot \mathrm{SD}^2}{\mathrm{~d}^2}=304 \text { participants }$$

**Fig. 1:**
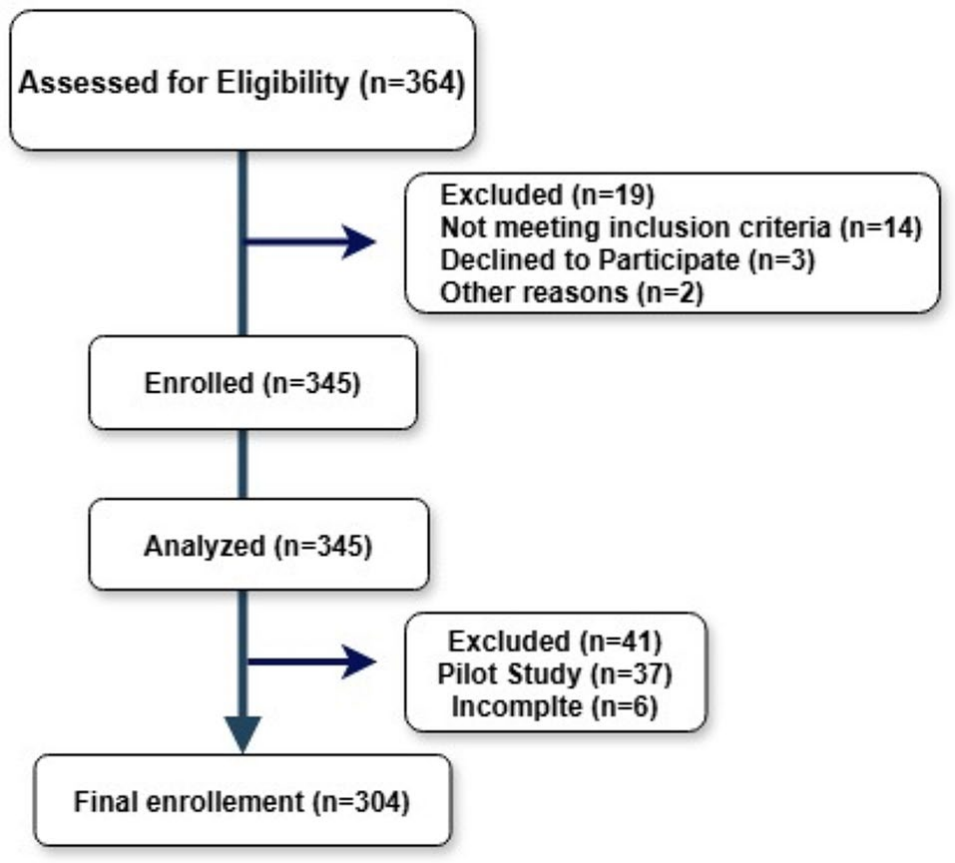
Participants enrollment diagram

Where Z1-α/2 = is the standard normal variate, at 5% type 1 error it is 1.96, SD = standard deviation of variable, and d = absolute error or precision. Data was collected through an anonymous online questionnaire distributed via school networks using Google Forms, with participation facilitated by schoolteachers to ensure high response rates while maintaining confidentiality and minimizing response bias (Fig. 1).

### Participants

This study recruited 304 preparatory and secondary school students (126 boys [41.4%] and 178 girls [58.6%]) aged 12–20 years (M = 15.3, SD = 1.9) from governmental schools in the Dakahlia Governorate, Egypt, between December 2023 and March 2024. A purposive sampling method was employed based on accessibility and administrative approval, and students meeting the inclusion criteria being within the 12–20 age range, willing to complete all online questionnaires, and having provided informed online consent from their legal guardian were invited to participate. Questionnaires from students who did not meet these criteria or were unwilling to participate were excluded from the analysis.

### Study instruments and measures

For this study, an online self-administered questionnaire was used, delivered in the participants’ native language (Arabic). The anonymous survey tool was created using Google Forms and distributed to participants through teachers at the participating schools. The questionnaire included relevant items from existing cyberbullying scales, self-control scales, and moral identity questionnaires, based on previous studies. The research instrument consists of four primary sections. The first section includes the participant’s sociodemographic characteristics, such as age, gender, educational level, living status, place of residence and parents’ educational level. Each item was carefully chosen to paint a complete picture of the diverse demographic attributes of the participating students. This meticulous approach to data collection was intended to ensure that the information gathered was both high-quality and relevant, significantly enhancing the understanding of the research context and facilitating more rigorous analyses in future studies.

The second section of the research instrument was the Cyberbullying Scale (CBS), developed by Stewart and colleagues (2014) [26]. It is a self-report measure designed to assess cyberbullying behaviors. The CBS consists of 14 items rated on a 5-point Likert scale (0 = Never, 1 = Almost Never, 2 = Sometimes, 3 = Almost All the Time, 4 = All the Time), with a maximum total score of 56. CBS captures various aspects of cyberbullying, including sending hurtful messages (e.g., “I have sent mean or hurtful messages to someone online”), spreading rumors (e.g., “I have shared false information about someone through social media”), and online exclusion (e.g., “I have deliberately ignored or excluded someone in an online group”). Given its ability to measure different forms of online aggression. CBS is a valuable tool in nursing research for examining the psychological and behavioral impacts of cyberbullying, particularly among adolescents and young adults. Cyberbullying levels are categorized as low (< 50%), moderate (50–75%), and high (> 75%). The original CBS demonstrated excellent internal consistency, with a Cronbach’s alpha of 0.94, indicating strong reliability. Saman and colleagues, (2021) used the scale and reported good reliability with Internal consistency measured by Cronbach’s alpha reached 0.87 [27]. In the present study, internal consistency was assessed, yielding a satisfactory Cronbach’s alpha value of 0.85.

The third research instrument’s part was Self-Control Scale (SCS): SCS is a 36-item self-report scale developed by Tangney and colleagues, (2004) [28]. The SCS is used to assess an individual’s capacity for impulse regulation, goal-directed behavior, and emotional control. Participants rate their agreement with each statement on a 5-point Likert scale (1 = not at all; 5 = very much), with total scores ranging from 36 to 180, where higher scores indicate greater self-control. The scale evaluates key aspects of self-regulation, including impulse control (e.g., “I am good at resisting temptation”), goal-directed behavior (e.g., “I am able to work effectively toward long-term goals”), and emotional regulation (e.g., “I have trouble concentrating,” reverse-coded). It also incorporates items related to resisting temptation, habit-breaking, and delayed gratification, making it a comprehensive tool for understanding self-control in various behavioral and psychological contexts. Given its relevance to self-regulatory processes, the SCS is widely utilized in nursing research to explore its implications for health-related behaviors, patient adherence to treatment plans, and emotional resilience in clinical settings. Pilarska and Baumeister (2018) reported good reliability for the SCS, with Cronbach’s alpha of 0.87. In the present study, internal consistency was assessed, yielding an acceptable Cronbach’s alpha value of 0.85.

The fourth part of the research questionnaire was the Moral Identity Questionnaire (MIQ), developed by Black and Reynolds (2016) [29]. This instrument is designed to assess moral identity through its two core dimensions: moral integrity and moral self. The Moral Self dimension evaluates the importance individuals place on moral principles as central to their identity, while the Moral Integrity dimension assesses the extent to which individuals act in accordance with these moral principles. Sample items from the MIQ include statements like “I try hard to act honestly in most things I do” and “It is important for me to treat other people fairly” under the Moral Self domain, while the Moral Integrity domain includes items like “If no one is watching or will know, it does not matter if I do the right thing” and “I will go along with a group decision, even if I know it is morally wrong.” These items are rated on a Likert scale, allowing respondents to indicate their level of agreement.

The MIQ includes 20 items that measure two facets of moral identity: Moral Self and Moral Integrity. The MIQ demonstrates high internal consistency, with Cronbach’s alpha values ranging from .86 to .91 across studies, and good test-retest reliability, indicating stability in scores over time. In our study internal consistency reliability (Cronbach’s coefficient) was used to assess the reliability of the instrument and it provided an acceptable value (0.82 and 0.73) respectively. Respondents rate their agreement with each item on a 5-point Likert scale, ranging from ‘strongly disagree’ to ‘strongly agree’. The total scores range from 0 to 80. The total scores range from 0 to 80 which is divided into low-level scores less than 50%, moderate level scores between 50 and 75%, and high level above 75%.

### Procedure

Data collection commenced after obtaining ethical approval from the Faculty of Nursing, followed by the necessary approvals from the educational administration overseeing the selected schools. The study utilized standardized instruments, which were originally developed in English and required translation into Arabic. The translation process was conducted in two stages. First, the instruments were professionally translated by the Center for Specialized Languages at the Faculty of Arts, Helwan University. To ensure accuracy and conceptual equivalence, a committee-based approach to translation and back-translation was subsequently employed [30, 31]. The committee consisted of four experts: a nursing professor, a psychology professor from Egypt, a school nurse proficient in English with experience in healthcare settings, and a native English educator. Each member independently translated the scales in parallel, and their versions were synthesized into a final Arabic version that was unanimously approved. A panel of experts in psychology, psychiatry, and psychiatric nursing then assessed the validity and cultural relevance of the translated instruments for use with school students. Modifications were made as needed to align the content with the local customs of Egyptian students. The final questionnaire underwent a pilot study with 30 participants to confirm its validity and clarity before implementation.

The survey was successfully administered to participants in schools through their teachers. The research instrument was developed using Google Forms and distributed via teachers, who provided students with an initial invitation containing a QR code or an online link to access the survey. The survey comprised two sections, with the first section serving as the informed consent form, which detailed the study’s objectives, confidentiality assurances, and participants’ right to withdraw at any time. To ensure ethical compliance, the informed consent was integrated into the research tool, requiring participants aged 13 and above to electronically confirm their consent before proceeding. For participants under the age of 13, parental or legal guardian approval was mandatory before participation. Access to the survey was granted only after the guardian’s electronic consent was obtained. Participants accessed the research instrument by scanning the QR code or using the provided hyperlink. All responses were automatically collected and securely stored in an Excel file, ensuring participant anonymity. The survey was primarily conducted via WhatsApp to enhance accessibility and facilitate participation.

### Statistical analysis

Data analysis was performed using SPSS 26.0 (IBM Inc., Chicago, IL, USA) to examine the survey responses from the recruited students. Descriptive statistics, including frequencies (percentages) and mean ± standard deviations (SD), were utilized to summarize both the general characteristics of the participants and the scores obtained on various scales. To determine the relative level of each variable (cyberbullying, self-control, and moral identity), we calculated the mean percent using the following formula:

$$\text { Maen Percent }=\left(\frac{\text { Mean score }}{\text { Maximum Possible score }}\right) X 100$$

This approach allows for standardized comparisons across variables by expressing the mean scores as percentages of their respective maximum possible scores. Higher mean percentages indicate greater levels of the respective construction. Parametric inferential statistics as t-test, (ANOVA) and regression analysis were used to examine the differences and similarities between study variables as well as analysis of variance to examine found correlations. Probability (p-value) of less than 0.05 was considered significant and less than 0.001 considered highly significant.

## Results

Our findings indicate that most of the students are adolescents, with 60.2% falling within the 14–16 age group and 58.6% being female. Regarding educational level, 34.5% of the students are in their third secondary year, while 21.7% are in their second secondary year. Additionally, 69.4% of the students reside in urban areas, and 94.4% report having a moderate living standard. Parental education levels reveal that 42.4% of fathers and 45.4% of mothers hold a bachelor’s degree or higher (see Table 1).

**Table 1:** Sociodemographic data of studied students (*n* = 304)

Sociodemographic data	No.	%
**Age**		
11–13	47	15.5
14–16	183	60.2
17+	74	24.3
Mean ± SD	15.3 ± 1.9	
**Gender**		
Female	178	58.6
Male	126	41.4
**Level of Education**		
First Preparatory	31	10.2
Second Preparatory	53	17.4
Third Preparatory	42	13.8
First Secondary	7	2.3
Second Secondary	66	21.7
Third Secondary	105	34.5
**Place of Residence**		
Urban	211	69.4
Rural	93	30.6
**Living Status**		
Low	2	0.7
Moderate	287	94.4
High	15	4.9
**Father Education**		
Illiterate	16	5.3
Read and write	25	8.2
Primary Education	18	5.9
Secondary Education	116	38.2
Bachelor or higher	129	42.4
**Mother Education**		
Illiterate	8	2.6
Read and write	137	45.1
Secondary Education	16	5.3
Bachelor or higher	138	45.4

Our findings reveal that 39.9% of the studied students reported experiencing cyberbullying, with a mean score of 25.5 out of a maximum of 64. In contrast, self-control levels averaged 113.1 out of 155 (73%), while moral identity was notably high, with a mean score of 81.8 out of 100 (81.8%) (see Table 2).

**Table 2:** Total scores of cyber bullying, Self-control and moral identity among the studied students (*n* = 304)

Items	Minimum	Maximum	Mean	Sd	Mean percent
Cyber bullying	16.0	64.0	25.55	10.80	39.9
Self-control	67.0	155.0	113.16	16.09	73.0
Moral identity	2.0	100.0	81.81	15.56	81.8

Table 3 shows a substantial 78.6% of students fall into the low cyberbullying category, with only 15.1% and 6.2% classified as moderate and high, respectively. For self-control, nearly all students exhibit at least moderate levels, with 58.6% moderate and 40.1% high, while only 1.3% show low self-control. Regarding moral identity, an impressive 78.6% of the students are at a high level, with 17.8% at a moderate level and only 3.6% at a low level.

**Table 3:** Levels of cyber bullying, self-control and moral identity among studied students (*n* = 304)

Levels	Cyber bullying		Self-control		Moral identity		
	No.	%	No.	%	No.	%	
Low	239	78.6	4	1.3	11	3.6	
Moderate	46	15.1	178	58.6	54	17.8	
High	19	6.2	122	40.1	239	78.6	

In our study the correlation analysis reveals that cyberbullying is significantly and inversely related to both self-control (*r* = -0.32, *p* < 0.001) and moral identity (*r* = -0.12, *p* = 0.02). This indicates that as self-control or moral identity increases, the incidence of cyberbullying decreases. Furthermore, a positive correlation between self-control and moral identity (*r* = 0.23, *p* < 0.001) suggests that these protective factors tend to reinforce each other (see Table 4).

**Table 4:** Correlation between cyber bullying, self-control and moral identity among studied students (*n* = 304)

Items	Cyber bullying		Self-control		Moral identity	
	*r*	*p*	*r*	*p*	*r*	*p*
Cyber bullying	1					
Self-control	-0.32	0.00*	1			
Moral identity	-0.12	0.02*	0.23	0.00*	1	

*Significant at p-value < 0.05

In our study the regression analysis demonstrates that higher self-control significantly predicts lower cyberbullying involvement (β = -0.287, *p* < 0.001). Although the direct effect of moral identity on cyberbullying is not statistically significant (β = 0.087, *p* = 0.087), the significant interaction between moral identity and gender (β = -0.221, *p* < 0.01) indicates that gender moderates the influence of moral identity on cyberbullying. This suggests that the protective role of moral identity may vary between male and female students. (see Table 5).

**Table 5:** Regression analysis for impact of self-control and moral identity on cyber bullying mediated by gender (*n* = 304)

Independent variables	Impact on cyber bullying			
	Regression coefficient	Standard error	t	*p*
Self-control	− 0.287	0.059	-4.832	0.000*
Moral identity	0.087	0.051	1.716	0.087
Gender	6.846	10.012	0.684	0.495
Self-control x gender	0.075	0.078	0.963	0.336
Moral identity x gender	− 0.221	0.079	-2.777-	0.006*

*Significant at p-value < 0.05

In our study, self-control remains a significant predictor of reduced cyberbullying behavior (β = -0.242, *p* < 0.001), confirming its critical role as a protective factor. In contrast, neither moral identity (β = -0.008, *p* = 0.833) nor living standard nor their interactions with the other variables show a significant effect on cyberbullying. These findings suggest that while self-control is a robust determinant of cyberbullying behavior regardless of socioeconomic status, the influence of moral identity does not vary significantly with living standard (see Table 6).

**Table 6:** Regression analysis for impact of self-control and moral identity on cyber bullying mediated by living standard (*n* = 304)

Independent variables	Impact on cyber bullying			
	Regression coefficient	Standard error	t	*p*
Self-control	− 0.242	0.039	-6.233-	0.000*
Moral identity	− 0.008-	0.039	− 0.211-	0.833
Living standard	12.846	25.933	0.495	0.621
Self-control x living standard	0.234	0.214	1.095	0.275
Moral identity x living standard	− 0.474	0.272	-1.745-	0.082

*Significant at p-value < 0.05

## Discussion

Social media usage presents both benefits and challenges. On the positive side, it facilitates access to educational resources and enhances social interactions [32]. However, excessive use may lead to negative consequences, including addiction and increased exposure to cyberbullying, a form of online harassment and intimidation. Among school-aged students, cyberbullying has been linked to levels of self-control and moral identity [33]. Strengthening self-control and fostering moral identity are essential strategies in mitigating cyberbullying, ultimately contributing to a safer and more supportive learning environment [34, 35]. Given the potential psychological and emotional impact of cyberbullying, nursing professionals and educators play a critical role in implementing preventive and intervention strategies to support students’ well-being.

Our study explored the prevalence of cyberbullying among Egyptian adolescents and its associations with self-control and moral identity, considering gender and socioeconomic factors. Findings revealed that 40% of participants had experienced cyberbullying, with an average score of 25.5 on a 64-point scale. These results align with global estimates, such as Patchin and Hinduja (2015), who reported a 34% prevalence among U.S. students [36], and Kowalski et al. (2014), who found rates ranging from 20 to 40% [37]. Similarly, Zhu et al. (2021) highlighted victimization rates between 13.99% and 57.5% [4]. While a Saudi Arabian study reported 42.8%, reflecting the cross-cultural prevalence of cyberbullying [38].

Our study found that students had an average self-control quotient of 113.1 out of a possible 155, indicating a commendably high level of self-control. This finding aligns with research by Fiddiana and Priyambodo (2022), who examined the correlation between self-control and cyberbullying in a private high school in Bogor. Their study reported that 74.7% of students exhibited high self-control, while 25.3% had moderate levels [39]. Similarly, our results are consistent with those of Vazsonyi et al. (2012), who found that adolescents with higher self-control were less likely to be involved in bullying, either as perpetrators or victims [40]. These studies suggest that self-control serves as a protective factor against cyberbullying behaviors.

Supporting this notion, Gámez-Guadix et al. (2015) reported that higher self-control is associated with lower levels of both cyberbullying and cyber victimization among adolescents, reinforcing its critical role in moderating such behaviors [41]. However, Brewer and Kerslake (2015) emphasized that while self-control is significant, other factors such as peer influence and online disinhibition also contribute to cyberbullying, suggesting that self-control alone may not be sufficient to prevent these behaviors [42].

Regarding the prevalence of cyberbullying, among the studied students, our study found that the majority reported low levels of cyberbullying, with fewer than one-fifth experiencing moderate levels. These findings align with Al-Adamat et al. (2023), who examined the predictive ability of moral identity for cyberbullying among university students and found that most students experienced low levels of cyberbullying [43]. This is further supported by Kowalski et al. (2014), who noted that while cyberbullying is widespread, its severity varies significantly among adolescents [37].

The findings of this study align with *Ecological Systems Theory (EST)*, offering a comprehensive understanding of cyberbullying as a multi-layered phenomenon shaped by individual, social, and environmental influences. At the microsystem level, self-control emerges as a crucial protective factor, directly regulating adolescents’ online behaviors and reducing cyberbullying tendencies. Moving to the mesosystem, the moderating effect of gender on moral identity highlights the role of socialization within families, peer interactions, and school environments in shaping ethical decision-making. Expanding outward, the exosystem findings suggest that while socioeconomic status does not significantly mediate cyberbullying, external environmental factors still influence adolescents’ digital experiences and access to online spaces.

At the macrosystem level, broader cultural and societal norms shape moral disengagement patterns, influencing how adolescents justify or reject cyberbullying behaviors. Finally, considering the chronosystem, the study underscores how the evolving digital landscape continuously reshapes cyberbullying risks, emphasizing the need for adaptive interventions. By framing these results within EST, this study extends the theory by demonstrating that addressing cyberbullying requires a multi-tiered approach, one that not only strengthens self-regulation at the individual level but also engages families, schools, and policymakers in fostering safer online environments for adolescents.

Additionally, Ang and Goh (2010) investigated cyberbullying in relation to affective and cognitive empathy, as well as gender, and found that the prevalence among boys and girls was 23.6% and 15.1%, respectively. Among males, 19.9% were classified as infrequent bullies, while 3.7% were frequent bullies. Among females, 14.2% were infrequent bullies, and 0.9% were frequent bullies [44]. More recent studies, such as Hinduja and Patchin (2019), further support these findings, suggesting that while cyberbullying remains a concern, many students experience it at lower severity levels [45]. The relatively low incidence of severe cyberbullying in the present study may reflect effective school policies or increased awareness of its consequences. Additionally, Athanasiou et al. (2018) conducted a cross-national study in seven European countries and reported that 13.3–37.3% of adolescents aged 14–17 years were victims of cyberbullying at moderate levels [46].

The present study revealed that 58.6% of students exhibited a moderate level of self-control, while 40.1% displayed a high level. These results are consistent with Rathakrishnan et al. (2023), who examined the interaction between academic stress and self-control in predicting psychological well-being and found that most students had moderate levels of self-control [47]. Additionally, Moffitt et al. (2011) reported that approximately 30% of adolescents exhibited high self-control, with the majority displaying low to moderate levels. The variation in findings may be attributed to demographic, cultural, or methodological differences in measuring self-control [48].

Regarding moral identity, our study found that more than three-quarters of the students had a high level, while less than one-fifth exhibited a moderate level. This suggests that most students possess a strong moral identity, potentially influenced by their engagement with various social and educational environments. These findings align with Melhem et al. (2020), who reported that most examined students exhibited a high level of moral identity [49]. Similarly, Onat and Kulaksizoğlu (2014) found that students in Turkey displayed medium to high levels of moral identity [50]. Additionally, Hardy et al. (2015) found that approximately 60% of students demonstrated high moral identity, while 30% exhibited moderate levels. Differences in cultural, educational, and familial influences may account for these variations [51]. However, our findings contradict those of Al-Adamat et al. (2023), who reported that students had moderate levels of moral identity and a low incidence of cyberbullying [43].

The present study revealed a significant inverse correlation between cyberbullying and both self-control and moral identity, indicating that students with higher self-control and moral identity were less likely to engage in cyberbullying. Additionally, a significant positive correlation was found between self-control and moral identity. These findings align with Li et al. (2023), who demonstrated that higher self-control reduces moral disengagement, subsequently decreasing cyberbullying incidents [16].

Similarly, a comprehensive review by Zhu et al. (2021) highlighted self-control and moral identity as critical protective factors against cyberbullying across various cultural contexts. The review emphasized that individuals with higher emotional intelligence and empathy—traits closely linked to moral identity—were less likely to engage in cyberbullying [4]. Pabian et al. (2015) further supported this by finding that adolescents with higher self-control were less likely to engage in cyberbullying [52]. Wright and Li (2013) also reported an inverse relationship between moral disengagement and cyberbullying, suggesting that students with strong moral identity, who internalize moral values, are less likely to engage in such behaviors [53]. Additionally, Gini et al. (2015) found a positive correlation between self-control and moral identity, suggesting that both traits contribute to prosocial behavior [54].

Regression analysis in the present study showed that self-control significantly and negatively influences cyberbullying. This finding is consistent with Whitten et al. (2021), who reported that lower self-control is a significant predictor of cyberbullying, particularly in environments that foster impulsive behaviors [55]. Holt et al. (2012) also demonstrated that self-control mediates the relationship between aggression and cyberbullying, indicating that enhancing self-control could help mitigate cyberbullying risks [56]. However, no significant relationship was found between moral identity and cyberbullying in the present study. This may be due to other psychological and social factors, such as moral disengagement, peer influence, and online anonymity, which may override the impact of moral identity. This finding aligns with Al-Adamat et al. (2023), who reported no significant relationship between cyberbullying and moral identity [43]. Conversely, Romera et al. (2021) found that moral disengagement had a medium-intensity positive correlation with cyberbullying, suggesting that higher moral disengagement increases the likelihood of cyberbullying behaviors [57].

The present study found that gender did not mediate the relationship between cyberbullying and self-control, indicating that both males and females were similarly influenced by self-control regarding cyberbullying behaviors. This finding aligns with Velensia et al., (2021) but contradicts Geng et al. (2022), who found that gender differences in emotional regulation could mediate this relationship [58, 59]. Furthermore, no significant differences were found in cyberbullying, self-control, and moral identity based on socioeconomic status. This contradicts Malinowska-Cieślik et al. (2023) and Vogel & Van Ham (2018), who found associations between socioeconomic disadvantage and aggressive behaviors [60, 61]. Finally, gender differences were observed in self-control and moral identity, with females scoring higher than males. This finding aligns with Jiamin (2023) but contradicts Tetering et al. (2020), who found no significant gender differences in self-regulation [62, 63].

## Conclusion

This study underscores the significant role of self-control in reducing cyberbullying among adolescents. Higher self-control was associated with lower cyberbullying involvement, making it a key protective factor. Although moral identity did not directly predict cyberbullying, its influence was moderated by gender, suggesting that its protective effects may vary between males and females. Socioeconomic status had no significant impact, reinforcing the importance of self-control across different demographic backgrounds. These findings highlight the need for targeted interventions to strengthen self-control and promote positive online behaviors among students.

### Implications for nursing practice

School and psychiatric mental health nurses play a critical role in mitigating cyberbullying by fostering self-control and moral development among students. Integrating structured interventions into school-based mental health programs can enhance students’ self-regulation and emotional control, reducing cyberbullying tendencies. Gender-sensitive strategies should be implemented to address differences in moral identity and their behavioral impact. Nurses should lead cyberbullying awareness initiatives, collaborate with educators and parents to establish a supportive digital environment, and implement early detection and counseling strategies to mitigate psychological consequences. These measures can strengthen students’ resilience and contribute to a safer, more positive school climate.

### Limitations of the study

Despite the importance of this study in identifying self-control as a key protective factor against cyberbullying, limitations should be acknowledged. Descriptive correlational design prevents causal inferences, self-reported measures may introduce bias due to potential overestimation or underestimation of experiences. Additionally, the findings are specific to the studied population, limiting generalizability to other cultural or educational settings. Future research should adopt longitudinal designs and diverse samples to strengthen these findings. Nonetheless, this study provides valuable insights for psychiatric and school nurses, emphasizing the need for interventions that enhance self-regulation skills to reduce cyberbullying and promote students’ psychosocial well-being.

## Data Availability

The corresponding author can provide the datasets used and/or analyzed for this study upon reasonable request.

## Abbreviations

CBS: Cyberbullying Scale
EST: Ecological Systems Theory
MIQ: Moral Identity Questionnair
SCS: Self-Control Scale
